# SHARESTART: A NEW PEDAGOGY TO PROMOTE LEARNING IN DIVERSE CLASSROOMS

**DOI:** 10.1093/geroni/igac059.035

**Published:** 2022-12-20

**Authors:** YanJhu Su

**Affiliations:** University of Massachusetts Boston, Boston, Massachusetts, United States

## Abstract

Diverse classrooms are nightly valued. The role of teachers is not limited to imparting knowledge. Nor are students only limited to gaining knowledge from their teachers. In this symposium session, the speaker would like to introduce a new pedagogy — Sharestart, which is specifically designed for comprehensive student learning. This pedagogy was proposed by a senior high school teacher in Taiwan. It is a long-term and stable teaching method that integrates a diversity of abilities by enabling students to self-learn, read, think, discuss, analyze, summarize, express and compose in every class session. In this symposium presentation, the speaker will introduce the core values of Sharestart pedagogy, discuss the advantages and disadvantages, and present an application scenario applying the Sharestart pedagogy.

